# Supplementary material for: Intermittent antibiotic treatment of bacterial biofilms favors the rapid evolution of resistance
Source: Commun Biol. 2023 Mar 16;6:275. doi: 10.1038/s42003-023-04601-y (PMC10020551; doi:10.1038/s42003-023-04601-y)
Supplement: Supplementary file 2 — Supplementary Material [file 42003_2023_4601_MOESM2_ESM.pdf]

SUPPLEMENTARY INFORMATION

Intermittent antibiotic treatment of bacterial biofilms favors the rapid evolution of resistance

Masaru Usui, Yutaka Yoshii, Stanislas Thiriet-Rupert, Jean-Marc Ghigo and Christophe Beloin*

This PDF file includes:
- Supplementary Note
- Supplementary Figures 1 to 14
- Supplementary Table S1 and S2

SUPPLEMENTARY NOTE

Mutations leading to moderate MIC increase in planktonic clones are diverse

The analysis of planktonic clones with enhanced MIC revealed diverse mutations associated or not with *sbmA* mutations (Fig. 5 and supplementary Table 4). Moderate MIC increase (20 to 24 µg/mL) in evolved planktonic clones were associated with mutations in *sbmA,* or in *cpxA*, the histidine kinase component of the *cpx* system known to be associated with increase resistance to aminoglycosides through regulation of the expression of porins and efflux pumps [7]. Mutations associated with higher MICs corresponded, in combination or not with *sbmA* alleles, to mutations in genes linked to aminoglycosides resistance via their capacity to modulate the proton motive force (PMF), notably genes encoding components of the Ubi complex responsible for the biosynthesis of ubiquinone (*ubiF, ubiH, ubiI, ubiJ*) [8, 9, 10] or genes belonging to NADH-quinone oxidoreductase Nuo (*nuoF*, *nuoG*)[11, 12, 13, 14]. We also identified a clone associating a *sbmA* mutation with a mutation in *aceF*, encoding a component of the pyruvate dehydrogenase complex potentially modulating the efficacy of aminoglycosides through modification of the PMF [15, 16], and with a mutation in *ribF* involved in riboflavin to FAD transformation that indirectly fuels electron transport chains. A mutation in the intergenic region between *gntX*, encoding a protein necessary for the use of DNA as a carbon source, and *nfuA*, encoding a Fe-S biogenesis protein also enhanced the MIC of a *sbmA* loss of function mutant. NfuA is known to help maturation of NuoG possibly explaining its potential link with resistance to amikacin [17].

Identified *fusA* mutations are biased towards domain IV and V pointing to a possible explanation of the observed resistance

All *fusA* mutations identified in our study were exclusively located in domains IV and V of the FusA protein. This therefore suggests that the amikacin treatment biased the selection of mutations towards these domains. Consistently, the alignment of all the *E. coli fusA* sequences

in the nrprot database of NCBI (2143 sequences) showed that domains I and V are the more variable but that it was not the case of domain IV (supplementary Fig. 13). Therefore, it is very unlikely that unbiased mutations be only found in domains IV and V in our evolved populations ($\chi 2$ test, p-value = 3e-06).

The elongating factor G domains IV and V are relatively rigid domains that move together with domain III during the pre- to post-translocation of charged tRNA from the A to the P-site of the ribosome [18]. During the translocation, domain V flips by 180° with a simultaneous 90° self-rotation causing domain IV to project into the decoding center of the 30S ribosome where the anticodon end of the A-site tRNA would be bound. Aminoglycosides bind the A-site decoding center, stabilize the interaction of tRNA in the A-site and ultimately inhibit elongation factor G-catalyzed translocation [19, 20, 21]. One can speculate that *fusA* mutations detected in our study could modify the dynamics of elongation factor G during translocation and/or its affinity with the A-site, thereby modifying binding of aminoglycosides to the decoding center of the 30S ribosome. Alternatively, but not mutually exclusive, such mutations could cause dysregulation of the expression of other factors leading to indirect effect on aminoglycosides activity, as recently shown for a mutation in the *fusA1* gene of *P. aeruginosa*, a hot-spot for mutations in patients with cystic fibrosis [22, 23].

SUPPLEMENTARY FIGURES

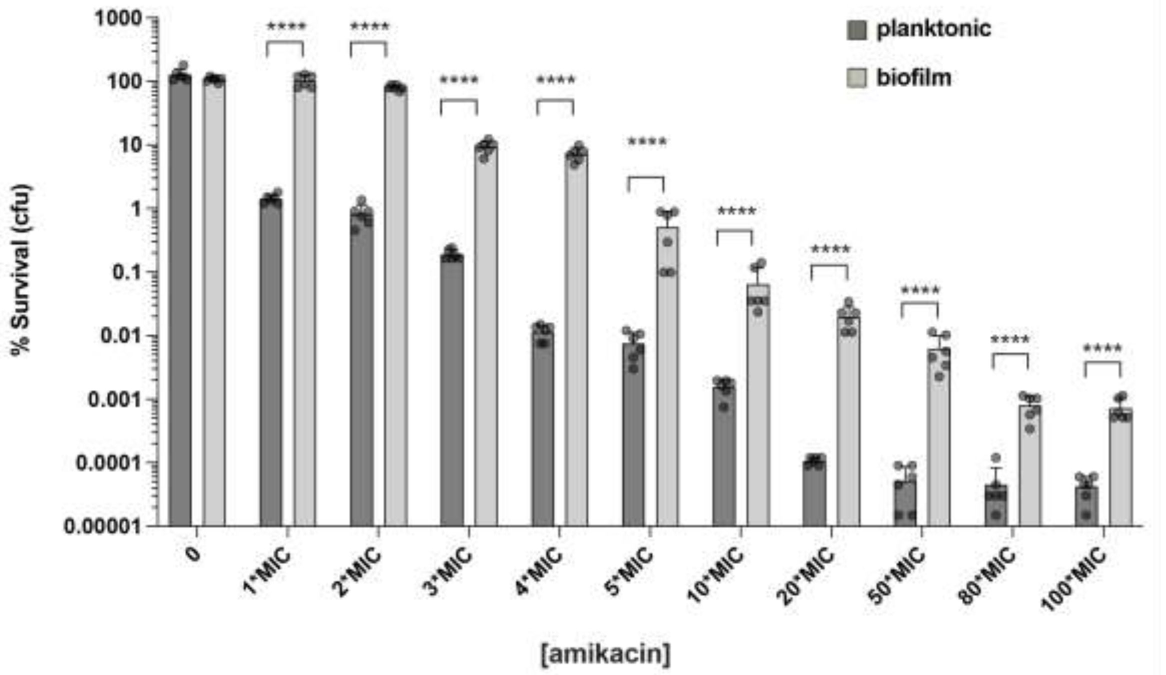

Supplementary Fig. 1. Amikacin concentration dependent killing of *E. coli* LF82 in planktonic and biofilm conditions. LF82 biofilms were grown on silicone coupons during 48 h at 37°C under static conditions in LB. LF82 planktonic bacteria were grown during 48 h at 37°C under agitation in LB. Biofilms (light grey) and planktonic cells (dark grey) were then exposed to increasing concentration of amikacin for 24h, after which silicone coupons or aliquots of planktonic cultures were collected, washed, serially diluted and plated for CFU counting and estimation of the survival rate. Each bar represents the mean of six biological replicates with standard error of the mean (n=6). Data were log10 transformed before being submitted to unpaired two-tailed t-test with Welch's correction. ****P ≤ 0.0001. From this concentration killing curve we could measure the amikacin Minimal Biofilm Inhibitory Concentration (MBIC) and the Minimal Biofilm Eradication Concentration (MBEC) of LF82 to be, respectively, 48 µg/mL and 160 µg/mL.

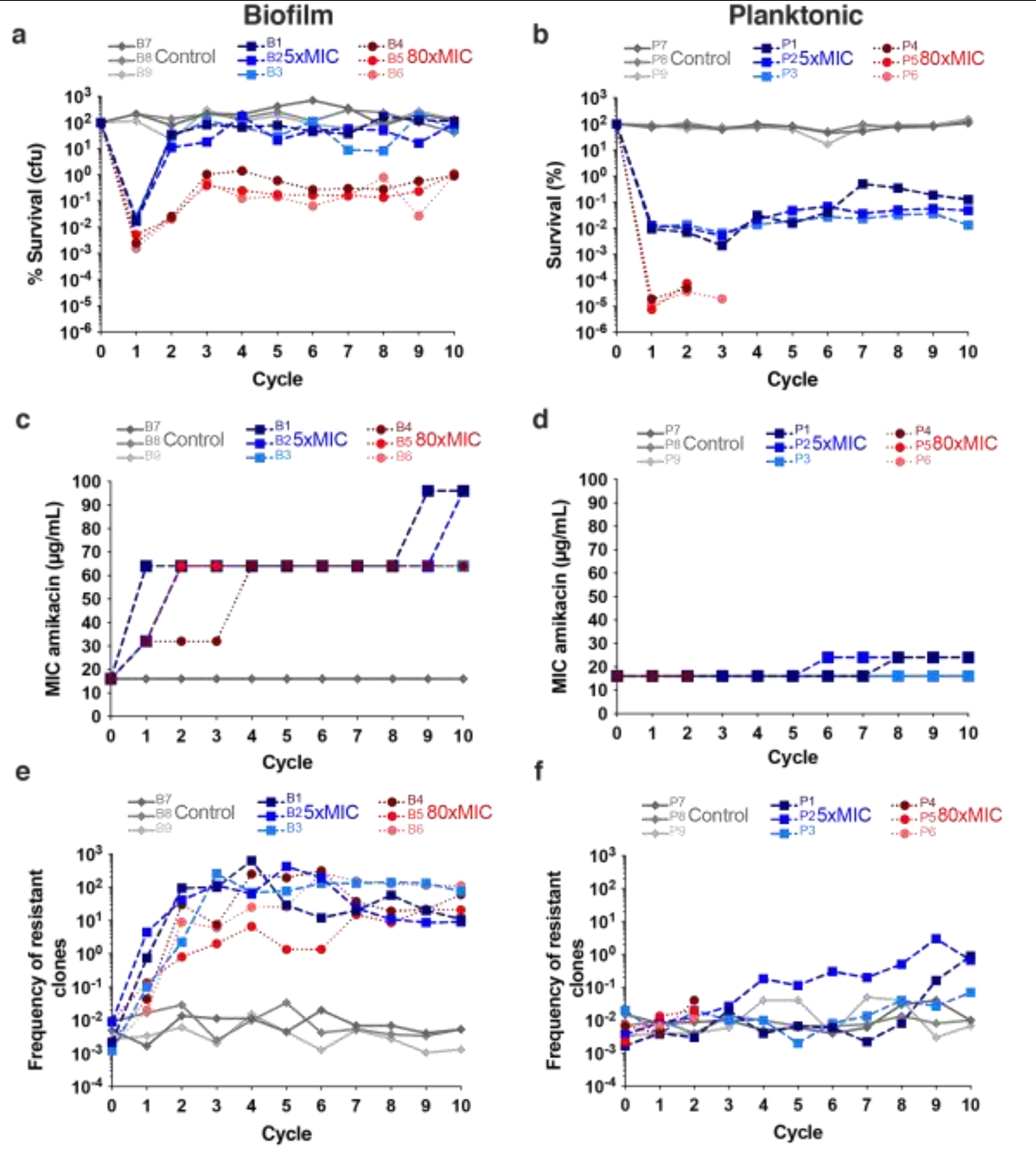

Supplementary Fig. 2. Evolution of *E. coli* under lethal antibiotic 24h intermittent treatment of amikacin as in Fig. 2 with independent evolution replicates plotted. Evolution in (a, c, e) Biofilms and (b, d, f) Planktonic**.** In (a, b) are represented the percentage of survival after each cycle of evolution at step 5 for biofilm and planktonic population as compared to population before each cycle of treatment at step 2 (see Figure 1). Percentages of survival were determined using CFU counting, $(CFU_{step5}/CFU_{step2})x100$. 100% ($10^2$ %) survival corresponds to no death after treatment. The three independent evolutions (n=3) were performed in parallel for each condition (no treatment= control in grey, 5x amikacin MIC in blue, 80x amikacin MIC in red). Values are represented on a log scale. In (c, d), the MIC to amikacin was determined in triplicate (n=3) for each population sampled at the end of each cycle using the agar dilution method. No variation of the MIC was observed between the triplicate. In (e, f), each population sampled at the end of each cycle was plated once on LB plate with or without 1x amikacin MIC (n=3) (for plating on 2xMIC and 4xMIC see supplementary Fig. 4). Frequency of resistant clones was calculated as the CFU_{1xMIC}/CFU_{LB}. Values are represented on a log scale. In (e, f), the representation is different from the one

of Fig. 2 to facilitate reading. In a to f, control in grey corresponds to the evolution where no antibiotic was added during step 3.

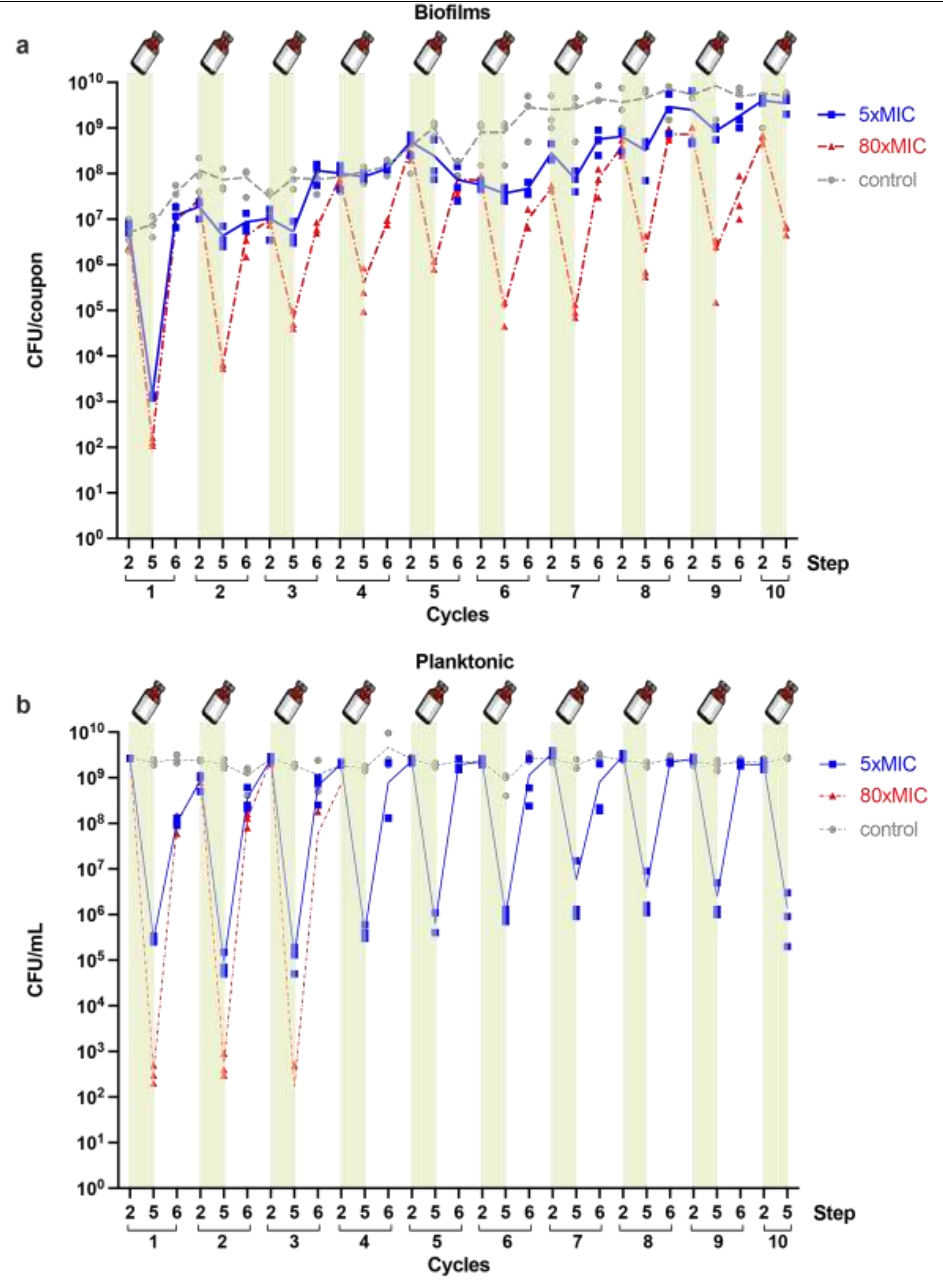

Supplementary Fig. 3. Evolution of colony forming units at each sampling step over the 10 cycles of the experiments, in Biofilm (a) and in Planktonic (b) population**.** CFU counting correspond to sampling for both biofilm and planktonic at steps 2, 5 and 6 for cycles 1−9 then steps 2 and 5 for cycle 10 (see Fig. 1). Three independent evolutions were performed in parallel for each condition (no treatment at step 3= control, 5x amikacin MIC, 80x amikacin MIC). At each step we represented the means of the three independent CFU

counts per coupon (biofilm) or mL (planktonic) (n=3) of the three experiments for control, 5x amikacin MIC, 80x amikacin MIC.

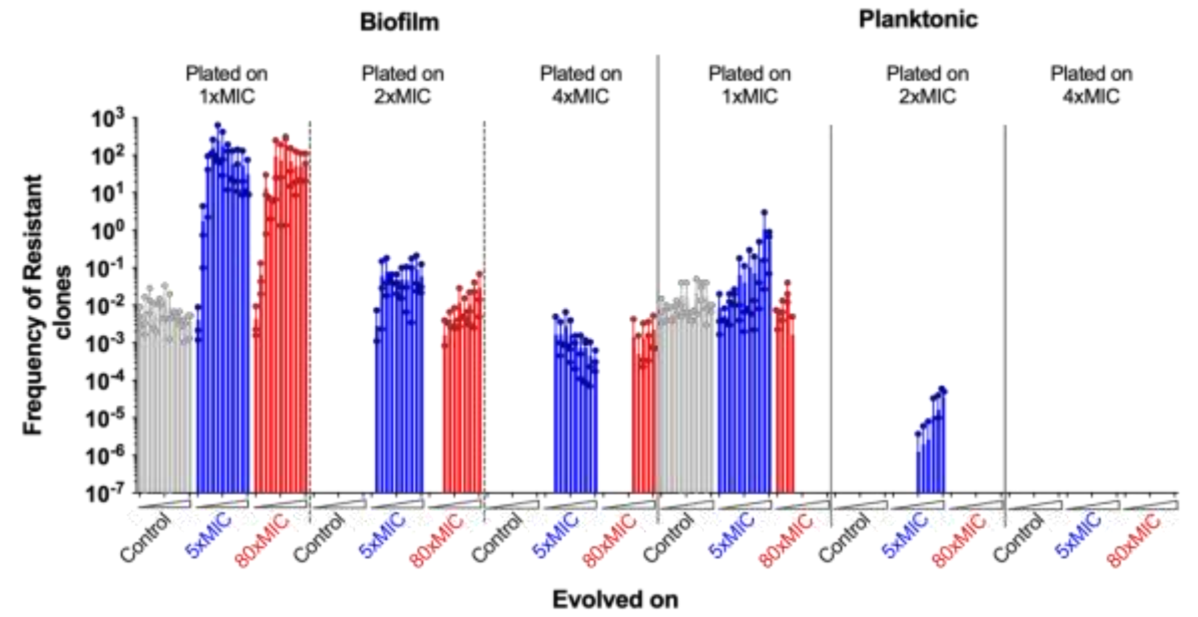

Supplementary Fig. 4. Frequency of resistant mutants growing on 1xMIC, 2xMIC or 4xMIC of amikacin over cycles of 24h intermittent treatment. Three independent evolutions were performed in parallel for each condition (no treatment = control, 5x amikacin MIC, 80x amikacin MIC). Values represented correspond to the means and standard errors of the mean of three measurements (n=3). Each 24h intermittently treated biofilm and planktonic population sampled at the end of each cycle was plated on LB plate with or without 1xMIC, 2xMIC or 4xMIC (1xMIC results are duplicated from Fig. 2e and f and presented here for comparison with 2xMIC and 4xMIC). Frequency of resistant mutants was calculated as the $CFU_{1xMIC\ or\ 2xMIC\ or\ 4xMIC}/CFU_{LB}$.

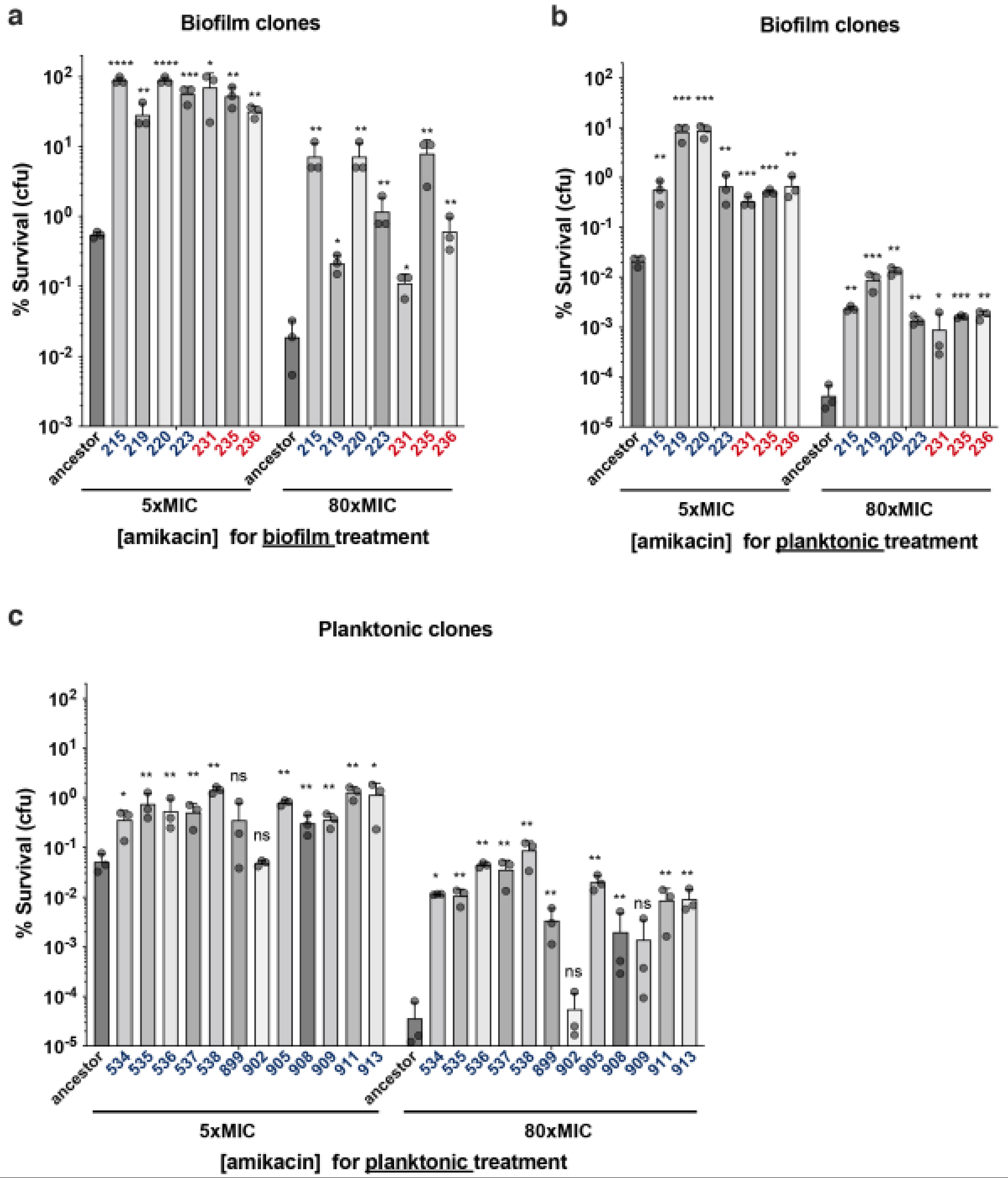

Supplementary Fig. 5. Survival of endpoint clones from biofilm and planktonic evolved populations. (a) % of survival of biofilm evolved cycle 10 clones when grown as biofilm on silicone coupons and treated by amikacin. Cycle 10 clones evolved in biofilms in presence of 5x amikacin MIC (in blue) or 80x amikacin MIC (in red) were grown as biofilm during 24h and treated or not for 24h by either of 5x amikacin MIC or 80x amikacin MIC and alive bacteria were quantified by counting colony forming units for each coupon. Survival was expressed as % of survival of the treated samples (n=3 biological replicates for each clone) as compared to the non-treated samples (n=3 biological replicates for each clone). (b) % of survival of biofilm evolved cycle 10 clones when grown as planktonic cultures and treated by amikacin. Cycle 10 clones evolved in biofilms in presence of 5x amikacin MIC (in blue) or 80x amikacin MIC were grown as stationary phase cultures and treated or not for 24h by either 5x amikacin MIC or 80x amikacin and alive bacteria were quantified by counting colony forming units. Survival was expressed as % of survival of the treated

samples (n=3 biological replicates for each clone) as compared to the non-treated samples (n=3 biological replicates for each clone). (c**)** % of survival of planktonic evolved cycle 10 clones when grown as planktonic cultures and treated by amikacin. Cycle 10 clones evolved in planktonic in presence of 5xMIC (in blue) of amikacin were grown as stationary phase cultures and treated or not for 24h by either 5xMIC or 80xMIC of amikacin and alive bacteria were quantified by counting colony forming units. Survival was expressed as % of survival of the treated samples (n=3 biological replicates for each clone) as compared to the non-treated samples (n=3 biological replicates for each clone). Data were log10 transformed before being submitted to multiple unpaired t tests with Welch correction. *P ≤ 0.05; **P ≤ 0.01; ***P ≤ 0.001, ****P ≤ 0.0001 and ns non-significant. These different clones have been sequenced and their associated mutations are described in supplementary Table 4.

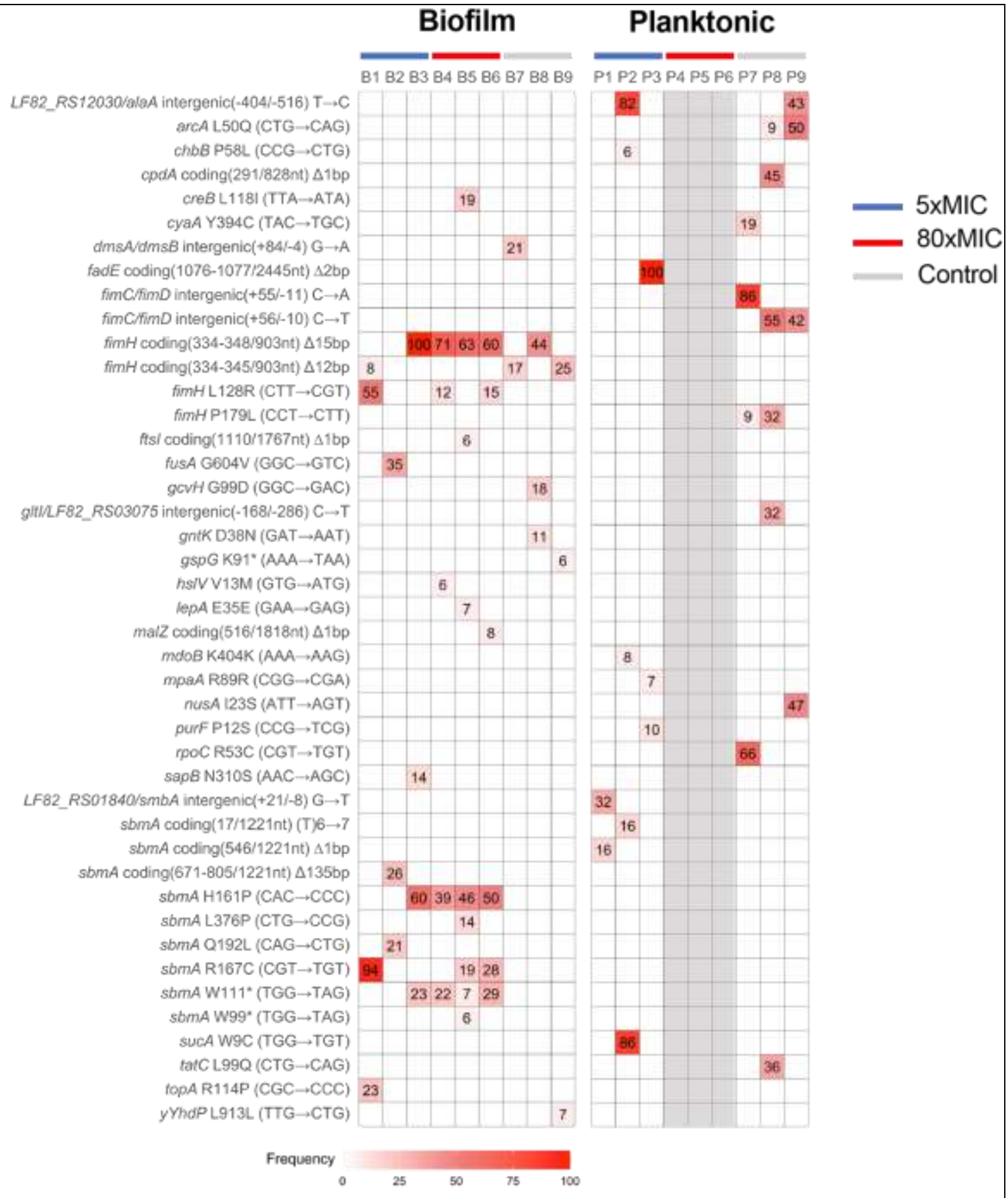

Supplementary Fig. 6. End-point population sequencing reveals a lifestyle associated pattern of mutations after evolution under antibiotic intermittent treatment (details at the alleles level). Mutations identified by whole-population genome sequencing of control and amikacin intermittently treated (5xMIC and 80xMIC) biofilm and planktonic populations of *E. coli*. The three populations corresponding to the three evolved lineages per lifestyle and treatment were sequenced after 10 cycles of treatment. The average depth of sequencing was x150. Mutations at higher frequency than 5% are detected by Breseq analysis. Red shading indicates the frequency of each independent mutation in the different locus at cycle 10 of the experimental evolution and the number in each box corresponds to the exact frequency detected. Population 4, 5, 6 from the planktonic lifestyle did not survive after 3 cycles and thus could not be sequenced at cycle 10 (they are shaded in grey). We therefore sequenced the population corresponding to the last cycle before their respective

extinction, but no mutation was detected at frequency of 5% or higher, with the exception of one fixed mutation in population P5 (see supplementary Table 2 and 3).

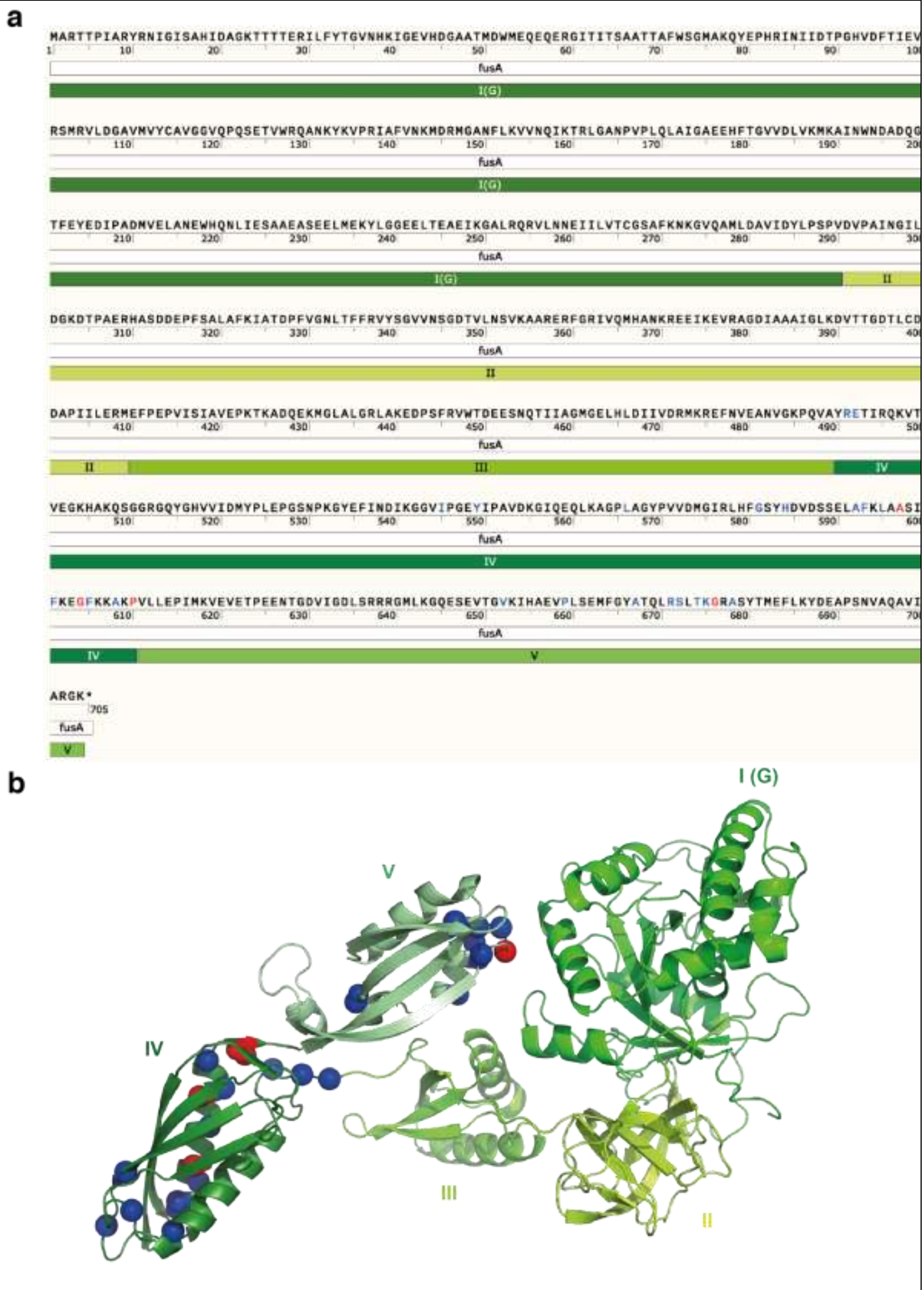

Supplementary Fig. 7. The elongation factor G (FusA) mutations leading to amikacin resistance. In (a) the primary sequence of *E. coli* LF82 FusA protein where the 5 functional domains are indicated. Amino-acids that correspond to positions that were identified as mutated and conferring enhanced amikacin MICs in this whole study are labeled in red for biofilm evolution on silicone coupons (A597V, G604V, P610L, G676C) and in blue for

planktonic evolution (R491C, E492Q, I545T, Y549N, L566P, G581S, H584Q, A592V, F593C, F593L, L595R, F601C, F601S, F601V, F605L, A608E, A608V, P610S, P610L, V652A, P659L, P659R, A667E, R671L, S672A, T674I, K675I, A678V). Mutations in blue were detected in clones only after concentrating planktonic evolved populations. In (b), the 5 functional domains and the different mutations are represented on the model structure of LF82 FusA protein as predicted using I-Tasser [24]. The same color code is used that in (a). The P610 position was mutated both in planktonic and in biofilm evolution.

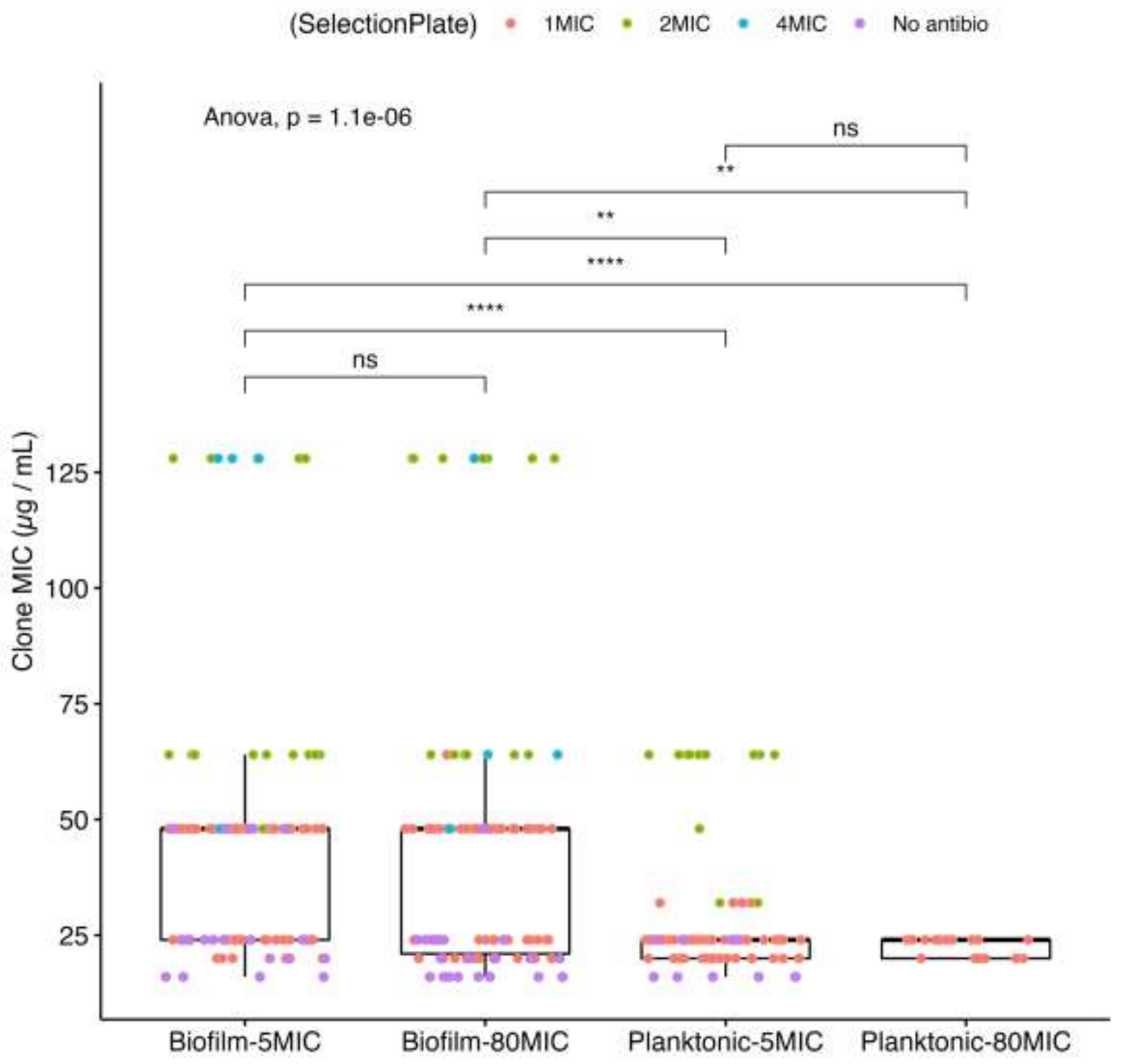

Supplementary Fig. 8. MIC of all clones isolated from biofilm and planktonic samples exposed to 5x and 80x amikacin MIC. The corresponding data are presented in supplementary Table 4, 5 and 6 (Biofilm-5MIC, n=102; Biofilm-80MIC, n=98; Planktonic-5MIC, n=74; Planktonic-80MIC, n=18)**.** For each condition the MIC for amikacin of each isolated clone is plotted, showing a higher mean MIC for the biofilm populations as compared to the planktonic populations (to one-way ANOVA followed by TukeyHSD post-hoc test). * p<0.05; ** p<0.01; *** p<0.001, **** p<0.0001. The plate on which each clone was isolated is indicated by a color code: purple on plates without antibiotic, red on plates with 1x amikacin MIC, green on plates with 2x amikacin MIC, blue on plates with 4x amikacin MIC. Statistical differences between pairs of conditions for each selection plate type were as follows (only significant ones are listed): no antibio (ns) ; 1MIC: Biofilm-5MIC vs Planktonic-5MIC, 6.7e-05; Biofilm-5MIC vs Planktonic-80MIC, 2.2e-03; Biofilm-80MIC vs Planktonic-5MIC, 5.2e-04; Biofilm-80MIC vs Planktonic-80MIC, 6.1e-03 ; 2MIC: Biofilm-80MIC vs Planktonic-5MIC, 0.007 ; 4MIC (ns).

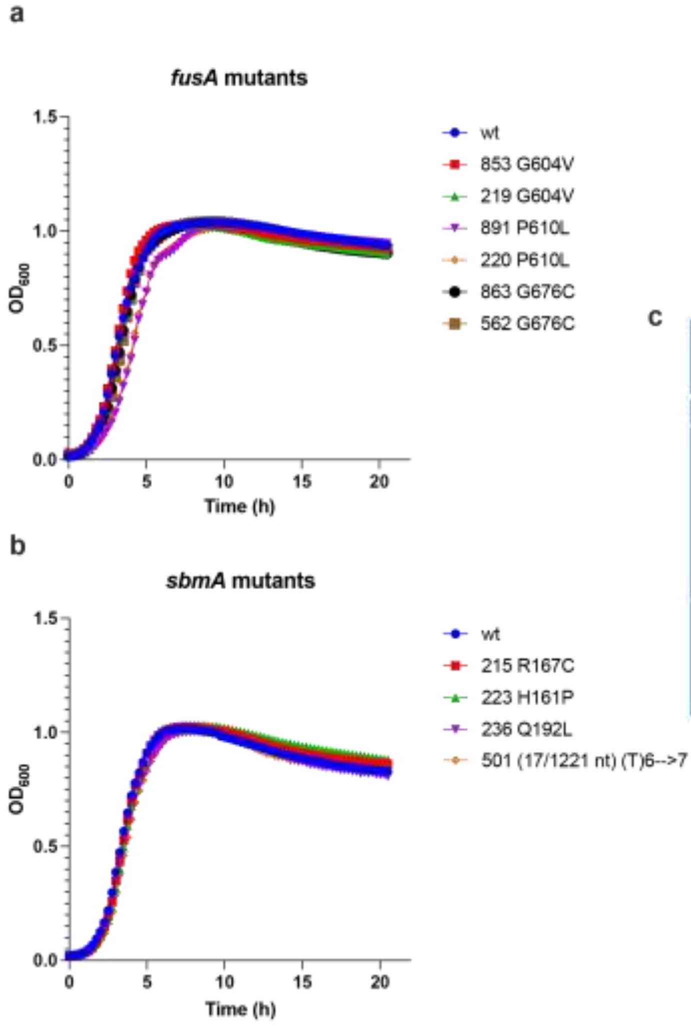

c

		Growth Rate (min^{-1})	Generation Time (min)	Carrying capacity (OD_{600})
	WT	0.0256	27.52	0.962
fusA	853	0.0261	26.55	0.987
	219	0.0274	25.34	0.974
	891	0.0196	35.28	0.989
	220	0.0200	34.68	0.983
	863	0.0251	27.60	0.975
	562	0.0249	27.87	0.993
sbmA	215	0.0276	25.09	0.946
	223	0.0271	25.60	0.967
	236	0.0253	27.49	0.915
	501	0.0268	25.84	0.913

Supplementary Fig. 9. Growth capacities of *fusA* and *sbmA* mutants. (a**)** 20h growth curve of *fusA* mutants in 96-well plate in LB medium. $FusA^{P610L}$ mutants (891 and 220) display slightly increased generation time compared to wt and other *fusA* mutants. (b) 20h growth curve of *sbmA* mutants in 96-well plate in LB medium, showing no difference among wt and *sbmA* mutants. For (a) and (b) growth curves of each strain were performed using 3 biological replicates. (c) Table showing the growth rate (min^{-1}), doubling time (min) and carrying capacities (OD_{600}) for all *smbA* and *fusA* mutants as inferred from the growth curves using the GrowthCurver R package [5]. It should be noted all these clones differ from the ancestral strain by a single *fusA* or *sbmA* mutation (see supplementary Table 4) and are otherwise isogenic.

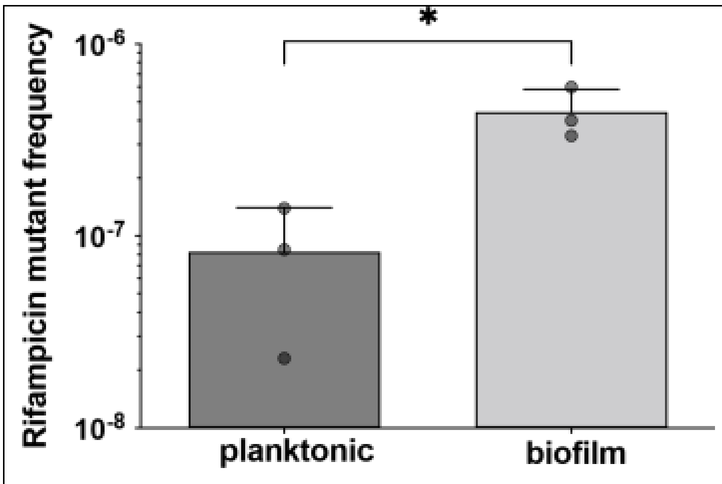

Supplementary Fig. 10. Biofilms on silicone coupons display higher mutation rate than their planktonic counterpart. Biofilms of LF82 strain (n=3 biological replicates) were grown on silicone coupon during 24h at 37°C under static conditions while planktonic cells (n=3 biological replicates) were grown under shaking conditions. After 24h of growth, silicone coupons or aliquots of planktonic culture were collected and plated on LB with or without 4xMIC of rifampicin. Frequency of resistant mutants was calculated as the CFU_{4xMIC}/CFU_{LB}. Statistical significance was evaluated using unpaired two-tailed t-test with Welch's correction. * $P<0.05$.

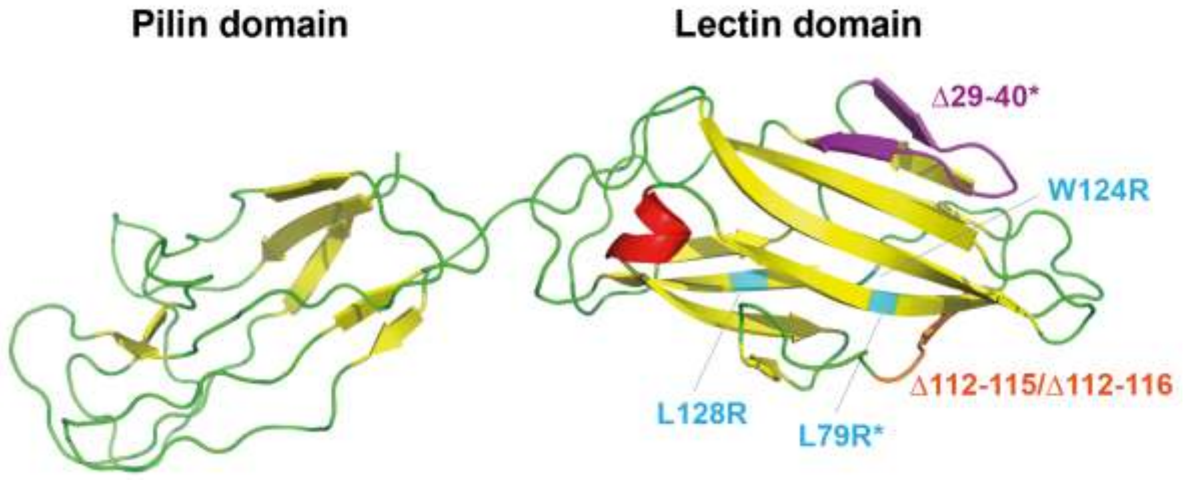

Supplementary Fig. 11. FimH mutations selected are in the lectin domain. Mutations identified in evolved biofilm populations are displayed on the LF82 FimH modeled 3D structure. The 3D structures of the mutant FimH proteins were predicted using the PHYRE2 Protein Fold Recognition Server [25]. All mutations are encompassed in the lectin domain. Mutations with a * corresponds to a in frame deletion (Δ29-40) or a mutated position (L79R) previously identified in our adaptive evolution experiments for increased adhesion and biofilm formation in *E. coli* K12 and for which we showed that there were gain of function mutations for enhanced biofilm formation [4].

A

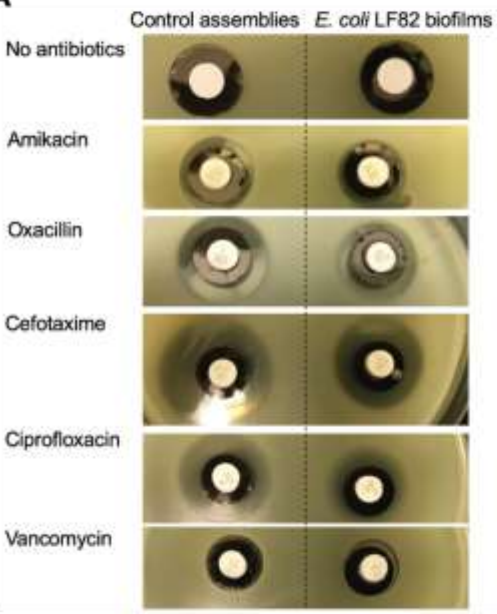

B

Antibiotic	Concentration (ug/ml)	Control assembly zone diameter (mm)	*E. coli* LF82 biofilms Zone diameter (mm)	% retardation	P value
No antibiotics		<13	<13	-	-
Amikacin	30	15.8 ± 0.29	14.3 ± 0.29	9.4 ± 3.0	S(P<0.05)
Oxacillin	1	20.7 ± 0.58	15.8 ± 0.76	23.4 ± 1.8	S(P<0.01)
Cefotaxime	30	27.7 ± 0.58	20.7 ± 0.58	25.3 ± 3.2	S(P<0.01)
Ciprofloxacin	5	20.5 ± 0.87	17.2 ± 0.58	16.2 ± 0.7	S(P<0.01)
Vancomycin	30	14.2 ± 0.29	<13	>8.2 ± 1.8	S(P<0.01)

Supplementary Fig. 12. Reduced penetration of antibiotics through LF82 biofilms. (A) Representative images of the penetration of antibiotics through the control assemblies and colony biofilms of *E. coli* LF82 as determined by the zone of *S. aureus* growth inhibition on MHA plates. (B) Averaged measurement of inhibition zones over triplicate experiments. The zones of growth inhibition (mean±SD) on MHA plates with control assemblies were taken as representative of 100% penetration and used to determine the percentage retardation of the penetration of antibiotics through biofilms. The limit of detection was 13 mm (size of the black, polycarbonate membrane filter on which biofilms were grown). S, significant. Two-tailed, paired *t*-test was used for statistical analysis.

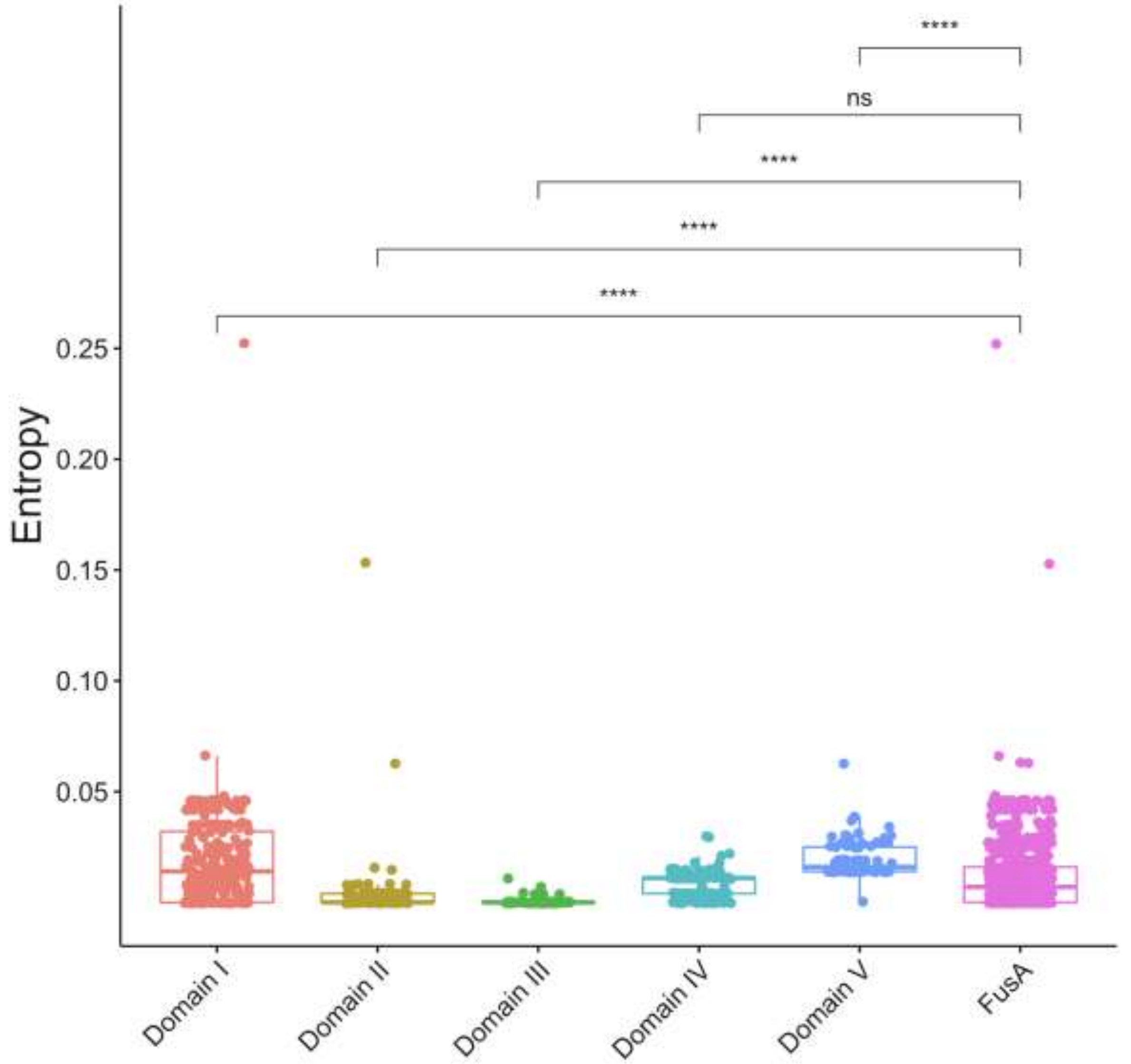

Supplementary Fig. 13. **Polymorphism level of different domains of the *E. coli* FusA protein.** Boxplots showing the entropy for each of the 5 domains of FusA as well as the whole FusA sequence within the multiple sequence alignment of all FusA protein sequences of *E. coli* in the nrprot database of NCBI (2143 sequences). The higher the entropy, the more variability in the alignment. Statistics correspond to one-way ANOVA followed by TukeyHSD post-hoc test comparing each domain to the whole *fusA* sequence. * p<0.05; ** p<0.01; *** p<0.001, **** p<0.0001. p values : Domain I vs FusA: 2.8e-6, Domain II vs FusA: 2.5e-6, Domain III vs FusA: <1e-7, Domain IV vs FusA: ns, Domain V vs FusA: 3.5e-4.

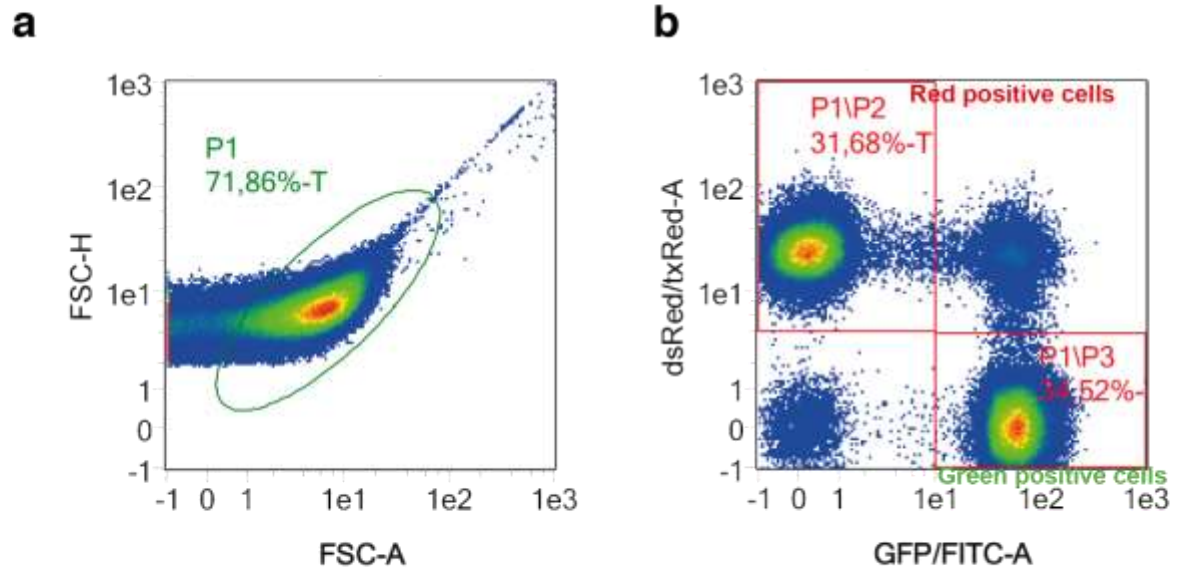

Supplementary Fig. 14. Gating strategy for flow cytometry data. (a) Gating was first performed on forward and side scatters (FSC and SC, respectively) to remove debris with triggers. Then, using FSC-Height (FSC-H) and FSC-Area (FSC-A) channels, cells along the diagonal were considered single cells and proceeded to the subsequent step. The singlets gate resulting from the exclusion of debris and doublets usually contained >70%. (b) Finally, the singlet cells were gated using GFP/FITC-A and dsRed/txRed-A channels and quantified for each color.

SUPPLEMENTARY TABLES

Supplementary Table 1. Strains and plasmids used in this study

**Strains**	**Description**	**Source**
LF82	AIEC LF82, isolated from a chronic ilea lesion from Chronic Disease patients, naturally ampicillin resistant	(Glasser *et al.*, 2001)
LF82 derivatives		
LF82_GFP	Δ*ampC*::gfp-FRT, deletion of *ampC* by gfp-KmFRT and removal of the kanamycin resistance using pCP20, GFP fluorescence	This study
LF82_mars	Δ*ampC*::mars-FRT, deletion of *ampC* by mars-KmFRT and removal of the kanamycin resistance using pCP20, mars fluorescence	This study
LF82_mars derivatives		
wt*fusA*-CmFRT	CmFRT from strain MG1655Δ*yfcV-P*::CmFRT placed after the *tufA* gene of LF82_mars, Cm^R	This study
Clone 219	LF82_mars, *fusA*_G604V, *fimH_L79R, LF82_RS22000_I255I*	This study
wt*fusA*-CmFRT, *fimH*_L79R	Deconstruction in clone 219 of *fusA* G604V by the region wt*fusA*-CmFRT of strain LF82_mars, wt*fusA*-CmFRT, Cm^R	This study
E. coli K-12 strains		
MG1655Δ*yfcV-P*::CmFRT	*yfcV-P* operon deleted by Cm-FRT cassette, Cm^R	(Korea *et al.*, 2010)
**Plasmids**		
pZE1R-GFP	Plasmid containing GFP under control of the phage λ *PcL* promoter. GFP is constitutively express in the cytoplasm of bacteria. Amp^R	(Lutz & Bujard, 1997)
pKD4	Pir dependent plasmid containing Km-FRT cassette, Km^R, Amp^R	(Datsenko & Wanner, 2000)

pZS*-2R-mars	Plasmid containing mars under control of the phage λ *PcL* promoter. mars is constitutively express in the cytoplasm of bacteria. Km^R	(Guet *et al.*, 2008)
pZE1R-GFP-KmFRT	Cloning of KmFRT from pKD4 into pZE1R-GFP, Amp^R, Km^R	This study
pZE1R-mars-KmFRT	Cloning mars open reading frame from pZS*2R-mars into pZE1R-GFP-KmFRT, Amp^R, Km^R	This study
pKOBEG	pSC101 ts, araC arabinose-inducible λ redγβα operon, Cm^R	(Chaveroche *et al.*, 2000)
pKOBEGA	pSC101 ts, araC arabinose-inducible λ redγβα operon, Amp^R	(Chaveroche *et al.*, 2000)
pCP20	Yeast Flp recombinase gene, Amp^R, Cm^R	(Cherepanov & Wackernagel, 1995)

REFERENCES

Chaveroche, M.K., Ghigo, J.M., and d'Enfert, C. (2000) A rapid method for efficient gene replacement in the filamentous fungus Aspergillus nidulans. *Nucleic acids research* **28**: E97.

Cherepanov, P.P., and Wackernagel, W. (1995) Gene disruption in Escherichia coli: TcR and KmR cassettes with the option of Flp-catalyzed excision of the antibiotic-resistance determinant. *Gene* **158**: 9-14.

Datsenko, K.A., and Wanner, B.L. (2000) One-step inactivation of chromosomal genes in Escherichia coli K-12 using PCR products. *Proceedings of the National Academy of Sciences of the United States of America* **97**: 6640-6645.

Glasser, A.L., Boudeau, J., Barnich, N., Perruchot, M.H., Colombel, J.F., and Darfeuille-Michaud, A. (2001) Adherent invasive Escherichia coli strains from patients with Crohn's disease survive and replicate within macrophages without inducing host cell death. *Infection and immunity* **69**: 5529-5537.

Guet, C.C., Bruneaux, L., Min, T.L., Siegal-Gaskins, D., Figueroa, I., Emonet, T., and Cluzel, P. (2008) Minimally invasive determination of mRNA concentration in single living bacteria. *Nucleic acids research* **36**: e73.

Korea, C.G., Badouraly, R., Prevost, M.C., Ghigo, J.M., and Beloin, C. (2010) Escherichia coli K-12 possesses multiple cryptic but functional chaperone-usher fimbriae with distinct surface specificities. *Environmental microbiology* **12**: 1957-1977.

Lutz, R., and Bujard, H. (1997) Independent and tight regulation of transcriptional units in Escherichia coli via the LacR/O, the TetR/O and AraC/I1-I2 regulatory elements. *Nucleic acids research* **25**: 1203-1210.

Supplementary Table 2. Oligonucleotides used in this study

Oligonucleotide name	Sequence 5'-3'
Cloning KmFRT in pZE1RGFP	
HindIII-KmFRT-5	gcgAAGCTTGTGTAGGCTGGAGCTGCTTC
SalI-KmFRT-3	gcgGTCGACTTAGTTCCTATTCCGAAGTT
Insertion of gfp- and mars-KmFRT into LF82	
ampC-up-XFP-KmFRT-5	CCCACCTATGGCGGGCCGTTTTGTATGGAAACCAGACCCTCTCGAGGATAAATATCTAACACCGT
ampC-down-XFP-KmFRT-3	AAGCGGAGAAAAGGTCCGAAAATTCGGACCCGATGAAATTGCAGGTCGACTTAGTTCCTATTCC
KmFRT-verif-5	GGATTCATCGACTGTGGCCG
XFP-KmFRT.verif-3	TAGCAGCCCTTGCGCCCTGAGT
ampC-up-ext5	CTGATTGGTGTCGTTACAATCTA
ampC-down-ext3	GGAACTGCAGGCAACGACCAG
Insertion of CmFRT after *tufA*	
Up.tufA-CmFRT-5	GCTTCATTTGTCAGGCTTTTTTCTTATTTTTAATGCCGCACTTTAATTGTGTAGGCTGGAGCTGCTTCGAAGTTCC
Down.tufA-CmFRT-3	GTCCTACGCAGGAATTATTTACGTTTTACGAGAACCCTTCAATATGCATATGAATATCCTCCTTAGTTCC
cat.verif-5	ACGCAAGGCGACAAGGTGC
cat.verif-3	TACATTGAGCAACTGACTG
tufA-CmFRT-ext-5	CTAAAGTTCTGGGCTAATTACACG
tufA-CmFRT-ext-3	CAACTGGAACGACGCTGACC
Sanger sequencing of *fusA* and *sbmA* genes	
fusA-5	GGCTACTTAAATTGAACGCC
fusA-5_2	ATCAAAACCCGTCTGGGCG
fusA-3	GAGAGAGCACGGGACTTTGG
sbmA-5	CGATAAGAAGTTAGCAGGAGTGC
sbmA-3	TGACGCGCGACATTACTTCTC
