# Supplementary material for: Intermittent antibiotic treatment of bacterial biofilms favors the rapid evolution of resistance
Source: Commun Biol. 2023 Mar 16;6:275. doi: 10.1038/s42003-023-04601-y (PMC10020551; doi:10.1038/s42003-023-04601-y)
Supplement: Supplementary file 3 — Description of Additional Supplementary Files [file 42003_2023_4601_MOESM3_ESM.pdf]

Description of Additional Supplementary Files

File name: Supplementary Data 1
Description: Cumulative number of generation over evolution cycles of biofilm and planktonic populations

File name: Supplementary Data 2
Description: Mutations identified using Breseq in end-point evolved populations at a frequency higher than 5%

File name: Supplementary Data 3
Description: Non-synonymous mutations at a frequency higher than 5% identified using Breseq in populations from different cycles of evolution

File name: Supplementary Data 4
Description: Sequenced clones from different populations from various cycles of biofilm and planktonic evolution

File name: Supplementary Data 5
Description: SbmA and FusA Sanger sequenced clones in biofilm and planktonic evolution

File name: Supplementary Data 6
Description: SbmA and FusA Sanger sequenced clones from planktonic population after concentration

File name: Supplementary Data 7
Description: List of E. coli FusA proteins and related genomes in NCBI in which a mutation was identified at the same position as one of the selected mutations in our evolutions

File name: Supplementary Data 8
Description: Raw data of the different experiments of the manuscript
