# Supplementary material for: Intermittent antibiotic treatment of bacterial biofilms favors the rapid evolution of resistance
Source: Commun Biol. 2023 Mar 16;6:275. doi: 10.1038/s42003-023-04601-y (PMC10020551; doi:10.1038/s42003-023-04601-y)
Supplement: Supplementary file 12 — Reporting Summary [file 42003_2023_4601_MOESM12_ESM.pdf]

nature portfolio

Corresponding author(s): Christophe BELOIN COMMSBIO-22-1889-T

Last updated by author(s): Jun 17, 2022

Reporting Summary

Nature Portfolio wishes to improve the reproducibility of the work that we publish. This form provides structure for consistency and transparency in reporting. For further information on Nature Portfolio policies, see our Editorial Policies and the Editorial Policy Checklist.

Statistics

For all statistical analyses, confirm that the following items are present in the figure legend, table legend, main text, or Methods section.

n/a	Confirmed	
☐	☒	The exact sample size (n) for each experimental group/condition, given as a discrete number and unit of measurement
☐	☒	A statement on whether measurements were taken from distinct samples or whether the same sample was measured repeatedly
☐	☒	The statistical test(s) used AND whether they are one- or two-sided<br>*Only common tests should be described solely by name; describe more complex techniques in the Methods section.*
☒	☐	A description of all covariates tested
☐	☒	A description of any assumptions or corrections, such as tests of normality and adjustment for multiple comparisons
☐	☒	A full description of the statistical parameters including central tendency (e.g. means) or other basic estimates (e.g. regression coefficient) AND variation (e.g. standard deviation) or associated estimates of uncertainty (e.g. confidence intervals)
☒	☐	For null hypothesis testing, the test statistic (e.g. F, t, r) with confidence intervals, effect sizes, degrees of freedom and P value noted<br>*Give P values as exact values whenever suitable.*
☒	☐	For Bayesian analysis, information on the choice of priors and Markov chain Monte Carlo settings
☒	☐	For hierarchical and complex designs, identification of the appropriate level for tests and full reporting of outcomes
☒	☐	Estimates of effect sizes (e.g. Cohen's d, Pearson's r), indicating how they were calculated

Our web collection on statistics for biologists contains articles on many of the points above.

Software and code

Policy information about availability of computer code

Data collection	nrprot database of NCBI (version 2019-04-09)
Data analysis	BreSeq version 0.35.0<br>lollipop tool (https://github.com/cdeitrick/Lolipop)<br>ggmuller R package 0.5.3 (Robert Noble (2019). ggmuller: Create Muller Plots of Evolutionary Dynamics; https://CRAN.R-project.org/package=ggmuller)<br>BLASTp (blast+ version 2.2.31)<br>mafft version 7.407<br>HIV sequence database website tool (https://www.hiv.lanl.gov/content/sequence/ENTROPY/entropy.html)<br>R package GrowthCurver<br>MACSQuantify™ Software (Miltenyi Biotec)

For manuscripts utilizing custom algorithms or software that are central to the research but not yet described in published literature, software must be made available to editors and reviewers. We strongly encourage code deposition in a community repository (e.g. GitHub). See the Nature Portfolio guidelines for submitting code & software for further information.

Data

Policy information about availability of data

All manuscripts must include a data availability statement. This statement should provide the following information, where applicable:

- Accession codes, unique identifiers, or web links for publicly available datasets
- A description of any restrictions on data availability
- For clinical datasets or third party data, please ensure that the statement adheres to our policy

DATA AVAILABILITY
All data of this study are included in this published article (and its Supplementary information File). All sequencing reads were deposited in NCBI under the BioProject accession number PRJNA833264. All source data underlying the graphs presented in the main figures are available as Supplementary Data.

Human research participants

Policy information about studies involving human research participants and Sex and Gender in Research.

Reporting on sex and gender	NA
Population characteristics	NA
Recruitment	NA
Ethics oversight	NA

Note that full information on the approval of the study protocol must also be provided in the manuscript.

Field-specific reporting

Please select the one below that is the best fit for your research. If you are not sure, read the appropriate sections before making your selection.

☒ Life sciences ☐ Behavioural & social sciences ☐ Ecological, evolutionary & environmental sciences

For a reference copy of the document with all sections, see nature.com/documents/nr-reporting-summary-flat.pdf

Life sciences study design

All studies must disclose on these points even when the disclosure is negative.

Sample size	no sample-size calculation was performed
Data exclusions	no data were excluded
Replication	A minimal of three biological replicates were generated providing variance of the results. Results were validated using statistical analyses.
Randomization	NA
Blinding	NA

Reporting for specific materials, systems and methods

We require information from authors about some types of materials, experimental systems and methods used in many studies. Here, indicate whether each material, system or method listed is relevant to your study. If you are not sure if a list item applies to your research, read the appropriate section before selecting a response.

Materials & experimental systems

n/a	Involved in the study
☒	☐ Antibodies
☒	☐ Eukaryotic cell lines
☒	☐ Palaeontology and archaeology
☒	☐ Animals and other organisms
☒	☐ Clinical data
☒	☐ Dual use research of concern

Methods

n/a	Involved in the study
☒	☐ ChIP-seq
☐	☒ Flow cytometry
☒	☐ MRI-based neuroimaging

Flow Cytometry

Plots

Confirm that:

☒ The axis labels state the marker and fluorochrome used (e.g. CD4-FITC).

☒ The axis scales are clearly visible. Include numbers along axes only for bottom left plot of group (a 'group' is an analysis of identical markers).

☒ All plots are contour plots with outliers or pseudocolor plots.

☐ A numerical value for number of cells or percentage (with statistics) is provided.

Methodology

Sample preparation	Bacterial cultures obtained from the competition assay experiment were diluted 300-fold with PBS in a microtube. Then, 200 µL of each diluted sample was inoculated in a 96-well plate for the flow cytometry analysis
Instrument	MACSQuant VYB flow cytometer (Miltenyi Biotec, Bergisch Gladbach, Germany)
Software	MACSQuantify™ Software (Miltenyi Biotec)
Cell population abundance	At least 10,000 events per sample were acquired. An abundance of cells gated as 'red- positive' or 'green-positive' depended on competition combinations.
Gating strategy	Gating was first performed on forward and side scatters (FSC and SC, respectively) to remove debris with triggers. Then, using FSC-Height (FSC-H) and FSC-Area (FSC-A) channels, cells along the diagonal were considered single cells and proceeded to the subsequent step (Supplementary Fig. 13A). The singlets gate resulting from the exclusion of debris and doublets usually contained >70%. Finally, the singlet cells were gated using GFP/FITC-A and dsRed/txRed-A channels and quantified for each color, as shown in Supplementary Fig. 13B.

☒ Tick this box to confirm that a figure exemplifying the gating strategy is provided in the Supplementary Information.
